# A New Era of Sustainability: Plant Biostimulants

**DOI:** 10.3390/ijms242216329

**Published:** 2023-11-15

**Authors:** Giuseppe Mannino

**Affiliations:** Plant Physiology Unit, Department of Life Sciences and Systems Biology, University of Turin, Via Quarello 15/A, 10135 Turin, Italy; giuseppe.mannino@unito.it

Today, environmental sustainability has become a fundamental concern in nearly every aspect of our daily lives, including the food sector. Food sustainability is deeply rooted, and continuously aiming to achieve it has compelling reasons. It not only ensures the quality of the food we consume but also guarantees the use of safe and environmentally sustainable practices. Consequently, food sustainability must start from the earliest stages of the production–consumption chain [1].

In this context, the role of plant biostimulants has emerged as a beacon of hope in agriculture, offering the opportunity to partially replace agricultural practices that have been used for decades and have now proven to be harmful and dangerous, not only for man health but also for the environment [2,3]. On the other hand, we need to realize that the quest for sustainable agriculture has never been more urgent. To confront the challenges associated with global population growth, the looming threat of climate change, and the depletion of finite resources, the imperative is clear: “We must maximize crop yields while minimizing the ecological footprint’’.

In this context, plant biostimulants have evolved into a revolutionary tool, embraced not only by farmers and researchers but also by policymakers. Indeed, in recent years, a concerted effort has materialized to promote research on biostimulants and foster their industrial development through substantial financial support and national policy directives. These products align seamlessly with the United Nations Sustainable Development Goals (SDGs), notably Goals 2 (Zero Hunger) and 15 (Life on Land) [4,5]. They also resonate with the European Union’s Green Deal and Farm to Fork strategy, both of which underscore the significance of sustainable agricultural practices [6,7,8]. We stand on the eve of a new era, in which the innovative use of biostimulants promises to reshape the way we cultivate crops and nourish our planet.

Plant biostimulants are natural or biologically derived substances that enhance plant growth, nutrient absorption, and stress tolerance. They are not fertilizers and do not replace the need for essential nutrients [9,10,11]. As the articles in this Special Issue demonstrate, biostimulants act as catalysts, facilitating improved nutrient uptake, root development, and overall plant vigor. Biostimulants, whether in the form of compound mixtures, natural extracts, or individual molecules, can provide a sustainable approach to agricultural practices when properly formulated and applied. This reduces the reliance on chemical fertilizers while simultaneously enhancing crop resilience [11,12]. Their capacity to increase nutrient efficiency and reduce the necessity for synthetic pesticides aligns seamlessly with the principles of sustainable agriculture, thereby contributing to a more food-secure and ecologically balanced world. Furthermore, by promoting root development and stress resistance, biostimulants aid crops in thriving under challenging conditions, including drought, salinity, or diseases. These mechanisms, in turn, can help in curbing excessive water use, thereby supporting water conservation efforts.

Nonetheless, while the potential of plant biostimulants is substantial, and their positive impact on sustainable agriculture is clear, the journey toward their widespread adoption is not without its challenges. To truly unlock the transformative potential of biostimulants, we must address several critical factors that are often underestimated today. For example, (i) as many authors who contributed to this Special Issue highlight, one of the most pressing issues in the biostimulant industry is the lack of standardized regulations. Unlike traditional agricultural tools such as fertilizers and pesticides, which benefit from well-established regulatory frameworks, biostimulants often find themselves in a regulatory gray area. This regulatory ambiguity results in the complete absence of quality control by manufacturers, who introduce products to the market without stringent oversight. Europe has taken a significant step in addressing this problem with the introduction of the European Union Fertilising Products Regulation [13,14]. These regulations help establish clear definitions, quality standards, and safety guidelines for biostimulants, bolstering the confidence of farmers and consumers. The global expansion and harmonization of such regulations are crucial to ensuring the reliability and safety of biostimulant products. (ii) Another major concern revolves around scientific research, which plays a pivotal role in fully realizing the potential of plant biostimulants. Researchers must conduct comprehensive studies to elucidate the mechanisms of action of biostimulants independently of manufacturing companies, which currently have a strong financial interest in obtaining swift and positive results. Furthermore, long-term studies are indispensable for a holistic evaluation of the sustainability and environmental impact of biostimulant use, a facet that has remained largely unexamined to date [15]. A robust body of scientific evidence not only enhances the confidence of farmers but also assists in tailoring biostimulant solutions to diverse agricultural contexts and influencing policymaking. (iii) In addition to impactful awareness campaigns, which can significantly contribute to the dissemination of knowledge, educational programs and training initiatives are crucial for increasing the acceptance and effective use of biostimulants among farmers and larger agricultural enterprises. (iv) Lastly, biostimulants should be readily available to a wide range of farmers, from large-scale commercial operations to smallholders. This is a critical point, as the currently high cost of biostimulants is difficult to justify, especially considering that many of them are derived from by-products of the agri-food industry [16,17].

In conclusion, plant biostimulants mark the advent of a new era in sustainability. As we endeavor to meet the growing demands of food production while safeguarding the environment, biostimulants offer a ray of hope. They represent the fusion of innovation and sustainability, promising a future where we can nourish the world without compromising the well-being of the planet. It is the collective responsibility of all of us—researchers, policymakers, and farmers—to embrace this transformative power and ensure the continued blossoming of this new era of sustainability.

The Special Issue, entitled “A New Era of Sustainability: Plant Biostimulants”, has brought together a diverse array of research articles, each shedding light on the multifaceted potential of plant biostimulants in reshaping our approach to sustainable agriculture. The remarkable range of topics covered in these manuscripts underscores the extensive and promising opportunities presented by plant biostimulants. These insights not only pave the way for more sustainable and environmentally friendly agriculture but also inspire further exploration and innovation in this dynamic field, fostering a deeper understanding of the profound impact that biostimulants can have on the future of our food production and the health of our planet. As we continue to explore this exciting frontier, collaboration among researchers, policymakers, and agricultural practitioners becomes increasingly vital. Together, we can leverage the potential of plant biostimulants to ensure a prosperous and sustainable future for agriculture, where we meet the global demand for food while nurturing the planet that sustains us. The content of this Special Issue serves as a testament to the dedication and passion of those who strive to venture into this new era of agricultural sustainability. 

As a conclusive remark, We would like to express our sincere gratitude to all those who have significantly contributed to the success of this Special Issue. In particular, We wish to thank the authors of the articles for their valuable and innovative contributions, as well as the reviewers for their dedication in critically evaluating and enhancing the contributions. A heartfelt thank you also goes to all the members of the editorial committee, whose support and guidance played a crucial role in bringing together this collection of articles.

We would also like to recognize the contributions of all those who worked tirelessly behind the scenes to ensure the quality and coherence of this Special Issue. Their commitment was instrumental in the successful completion of this project. Finally, We acknowledge the contributions of those who facilitated the publication of this Special Issue and supported the advancement of research in our field. Thank you to everyone for making this Special Issue a reality and for sharing your knowledge and dedication with us.
